# Predicting Foam Characteristics of Body Wash Products Using Machine Learning

**DOI:** 10.1021/acsomega.6c03163

**Published:** 2026-10-24

**Authors:** Jonghun Lee, Sivaramakrishnan Swamyna, Akshay Adithya, Viktor Dubovoy, Paul Thomson, Detravious J. Brinkley, Chibuzor J. Okocha, Christan Grant

**Affiliations:** † Colgate-Palmolive Co., 909 River Rd., Piscataway, New Jersey 08854, United States; ‡ Department of Computer & Information Science & Engineering, 3463 University of Florida, 1889 Museum Road, Gainesville, Florida 32611, United States

## Abstract

Foam generation and structure strongly influence the consumer perception of body wash products, yet systematic optimization of foaming properties is challenging because formulations are complex multicomponent mixtures. Here, we develop machine learning (ML) models to predict key foam attributes of body wash formulations from detailed composition data. A data set of 545 proprietary formulations, comprising 362 distinct ingredients, was assembled by combining internal formulation records with foam measurements. Six foam descriptors were quantified: maximum foam volume, initial and final bubble count, initial and final mean bubble radius, and bubble count half-life. Five ML algorithms—decision tree, k-nearest neighbors, extra trees, gradient boosting machines, and random forest were trained and evaluated by stratified 5-fold cross-validation repeated with five random seeds. The results indicate that ensemble tree-based models outperformed the single-tree and k-nearest neighbor approaches, with random forest achieving cross-validated coefficients of determination (*R* ^2^) between 0.62 and 0.86 across the six attributes. Shapley additive explanations (SHAP) analysis identified sodium laureth sulfate, cocamidopropyl betaine, opacifiers, fatty acids, and xanthan gum as the most influential contributors to foam behavior. Finally, we demonstrate an ML formulation capability using the trained models to explore trade-offs between foam volume and foam density. This study illustrates how ML can accelerate formulation development and enable data-driven design of personal care products.

## Graphical abstract

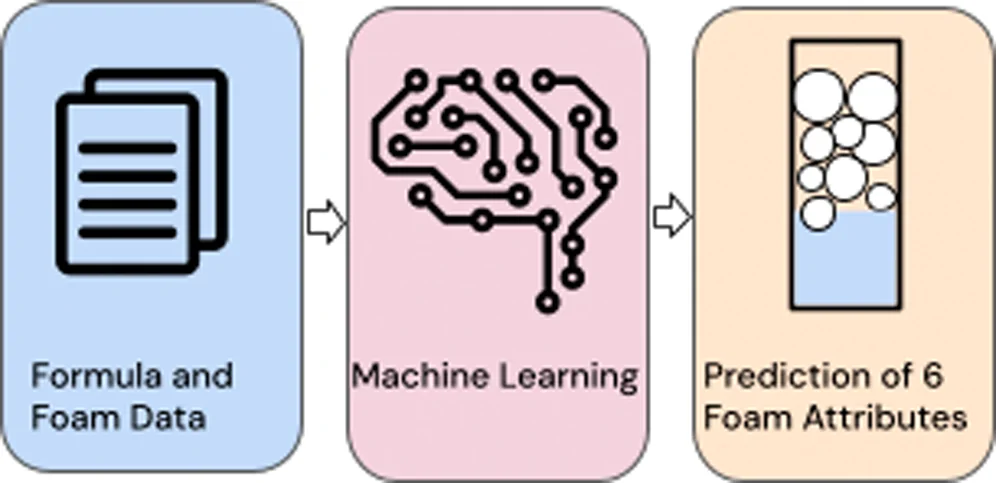

## 1 Introduction

For personal care products, delivering positive sensorial perception is critical as it strongly influences consumer purchase decisions. Foaming is one of the key attributes that consumers perceive during use and is often associated with effective cleansing.^1–3^ Although foam preference is subjective—for example, some consumers prioritize a creamy or luxurious texture, whereas others prefer large foam volume—the perception is ultimately linked to measurable physical properties of the foam that can be tuned through formulation. Foaming behavior is primarily governed by surfactants and polymers,^1,4,5^ but interactions with other components such as fragrance oils or solid particles can also significantly impact foam generation and stability.^6^ Because personal care products are complex multicomponent mixtures, fully understanding all ingredient–ingredient interactions and their collective impact on foam properties is challenging.

The consumer goods industry has traditionally relied on design-of-experiments (DOE) approaches to guide formulation optimization. DOE minimizes the number of required experiments by systematically varying the levels of a limited number of ingredients of interest. However, DOE still requires formulators to prepare and test each sample, and it becomes impractical with too many variables as the number of required experiments grows exponentially with the number of factors. Data-driven modeling via machine learning (ML) offers a promising route to accelerate formulation development, provided a sufficiently large and high-quality data set is available. In contrast to DOE, building ML models does not require balanced or orthogonal variation of ingredients, making them well suited for complex product spaces such as body washes, where ingredient usage patterns reflect historical formulation practice rather than controlled experimental design. In addition, the ML prediction based on the historical data significantly reduces the time and effort of scientists by eliminating the steps to prepare and test DOE samples. Because of this advantage, developing predictive ML models for formulation is gaining large momentum across personal care industries. Significant progress has been made across several domains, including ingredient discovery, sustainability, personalized skin care, toxicity and sensitization modeling, regulatory safety assessments, and the prediction of physicochemical properties.^7–17^ Recently, commercial artificial intelligence (AI) platforms have emerged to estimate macroscopic properties such as phase stability, surface tension, rheology, and foam. However, predictive formulation remains limited by the scarcity of high-quality, structured data.^17^ Furthermore, physical characteristics—particularly foaming behavior—are highly sensitive not only to composition but also to experimental conditions, such as dilution ratios, agitation dynamics, and cell geometry. Consequently, models trained on heterogeneous data often struggle to isolate true compositional effects. To the best of our knowledge, existing ML research on foam has primarily focused on the petroleum industry, specifically regarding foam rheology and half-life for enhanced oil recovery,^18,19^ leaving a significant gap in personal care applications.

To address this gap, we leveraged a comprehensive proprietary data set of 545 body wash formulations that include both experimental prototypes and globally commercialized products from Colgate-Palmolive—comprising 362 distinct active ingredients. To ensure real-world applicability, the complete, nonsimplified chemical compositions of the formulations were utilized for model training. Furthermore, we explicitly address the critical impact of testing protocols on foam characterization. By maintaining strictly standardized measurement protocols across all formulations under fixed dilution and agitation parameters, the models accurately isolate and resolve true compositional effects. Using the compositions of individual ingredients as input features, the models predict six quantitative foam responses: maximum foam volume, initial and final bubble counts, initial and final average bubble radii, and bubble count half-life. Five machine learning algorithms were benchmarked to identify the best performing prediction model. We discuss their predictive performance, and how Shapley additive explanations (SHAP) analysis can be leveraged to find the influential ingredients in foaming properties. Finally, we deploy the best-performing ML model as a visualization and “virtual recipe” application that allows formulators to rapidly explore formulation space, design new body wash formulas, and predict their foamability without the need for physical prototyping.

## 2 Methodology

### 2.1 Data Set and Formula Matrix Assembly

Historical formulation records for all evaluated samples were systematically retrieved from a proprietary internal database of Colgate-Palmolive Company. The compiled data set comprises 545 distinct, globally sourced body wash formulations, spanning both experimental prototypes and commercialized products. Across the entire data set, a total of 362 unique chemical ingredients were identified. To make this data accessible for machine learning pipelines, the raw formulation records were assembled into a structured tabular feature matrix. Each row in the matrix represents an individual product formulation, while each column represents a distinct ingredient feature. The values within the matrix correspond to the net active composition of each ingredient expressed as a weight percentage. Because any single commercial body wash utilizes only a finite subset of the total ingredient library, each formulation contains structural zeros for ingredients that are not included in that specific recipe. The resulting feature space is high-dimensional (362 descriptors) and inherently sparse where nearly 97% of the matrix is zero. Despite increasing risk of overfitting with high dimensionality, feature aggregation for dimensionality reduction is not considered in this study. Unlike pure chemistry research, ingredients in industrial formulations often behave differently across vendors despite sharing the same International Nomenclature of Cosmetic Ingredients (INCI) names. This variation likely stems from differences in manufacturing processes and impurity control. Instead, we rely on the structural regularization capabilities of tree-based ensembles discussed in Section 2.5 to manage this sparse data matrix.

### 2.2 Experimental Foam Measurement

Foaming properties of the body wash formulations were characterized using a Dynamic Foam Analyzer (DFA100 from Krüss). A borosilicate glass cylinder (inner diameter 40 mm, height 250 mm) equipped with a triangular prism on one side was used as the measurement cell. Figure 1 illustrates the foam measurement procedure. Samples were prepared by diluting each body wash to 5 wt % in deionized water. A 50 mL aliquot of the diluted sample was gently introduced along the cylinder wall to minimize premature foam formation. Foam was generated by six agitation cycles (stirring at 5000 rpm for 5 s, followed by a 10 s rest period) using a two-bladed stirrer (28 mm width). Intermittent foam volume was recorded during the rest period of each cycle. After the final agitation step, the foam was left undisturbed for 300 s to assess foam stability. The camera was positioned just below the foam–air interface, and during the 300 s rest period, bubble counts and mean bubble radius were measured every 0.5 s. For most formulations, three replicate measurements were performed, and the resulting parameters were averaged and used as responses to the machine learning models.

**Figure 1:**
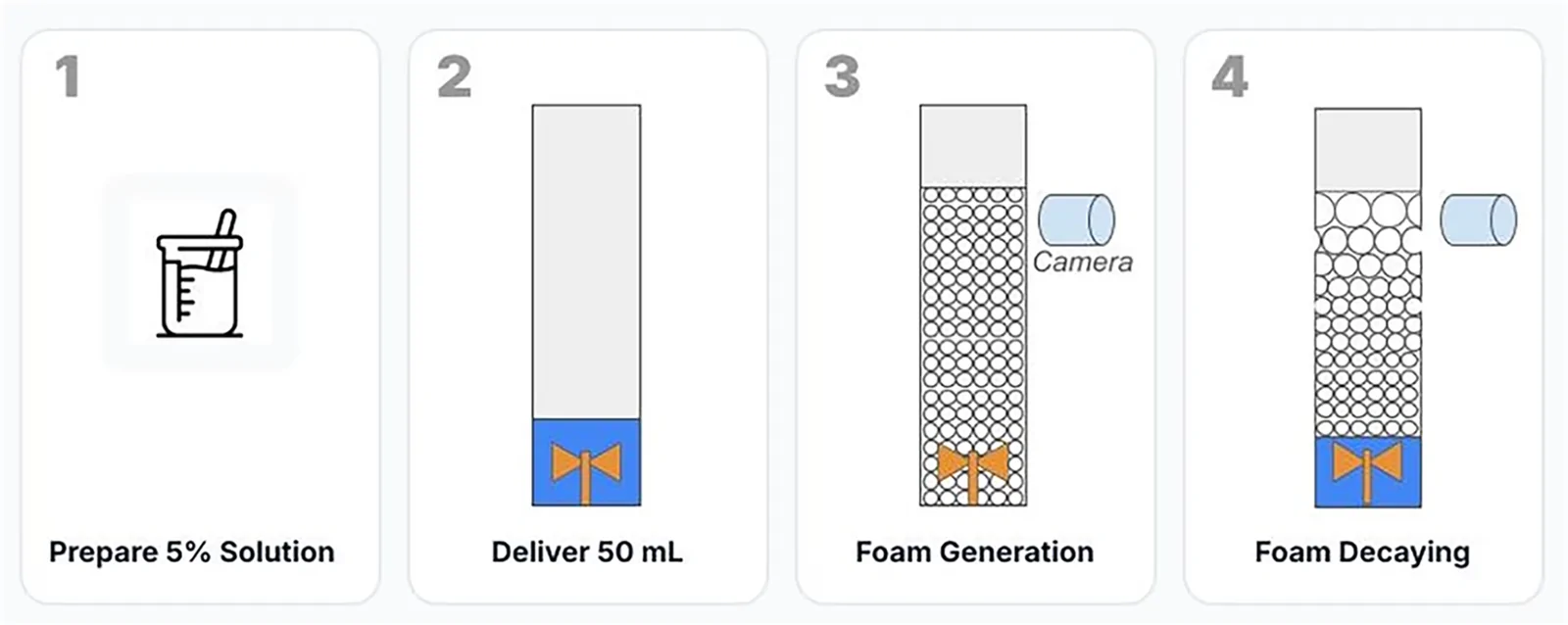
Schematic representation of foam measurements.

### 2.3 Foam Volume Correction

Dynamic Foam Analyzer (DFA) measures foam volume by optical transmission and foam structure by imaging. While we used foam structure data as given by DFA, we manipulated the foam volume profile to address the artifact by the DFA measurement. When foam is generated in a cylinder, a transparent liquid layer forms at the bottom, an opaque foam layer occupies the middle region, and a transparent air layer forms at the top. DFA determines the liquid height at the foam-liquid interface and the total height (foam + liquid) at the air-foam interface. The difference between the two heights becomes foam height. The height values are converted to the corresponding volumes. However, as the stirrer partially blocks the light path on the bottom, DFA inaccurately records the liquid volume in the early regime, which appears as the flat liquid volume of ∼30 mL in the first ∼125 s in Figure 2. With aging of bubbles, water in foam drains down, which increases the liquid height. Only after the liquid height becomes higher than that of the stirrer, DFA is able to detect the liquid volume correctly, at which the liquid line in Figure 2 begins to increase. This false reading of liquid volume causes underestimation of the maximum foam volume. To correct the artifact, the liquid volume curve is fitted with an exponential function in the region where the measured liquid volume begins to rise and continues to its final value (eq ([Disp-formula eq1])).

1 $$\begin{array}{ll} {V_{liq,corrected}}_{\;}=0 & if\;t<t_{0}\\ V_{liq,corrected}=V_{liq,m}-V_{liq,m}\cdot e^{-k\left(t-t_{0}\right)} & if\;t<t_{0} \end{array}$$

**Figure 2:**
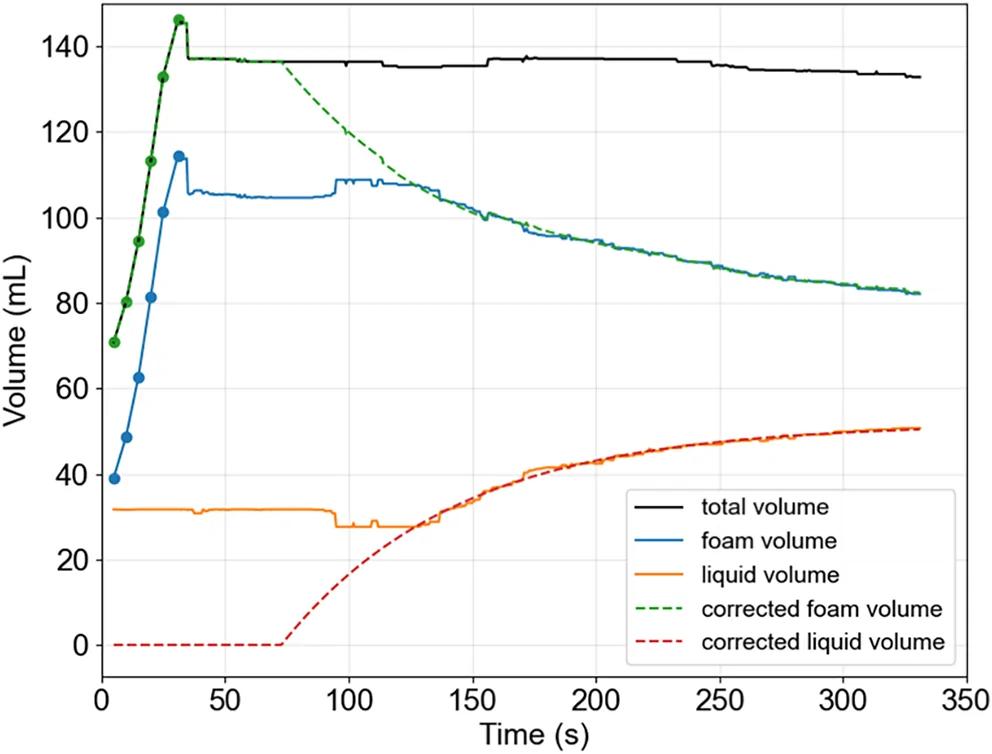
Foam and liquid volume profiles measured by Dynamic Foam Analyzer (solid lines) and after the correction (dashed lines).

where *V*_liq,corrected_ is the corrected liquid volume, *V*_liq,*m*_ is the fitted maximum liquid volume, *k* is a fitting parameter, *t*_0_ is the onset time at which liquid volume begins to rise from zero, and *t* is time. The foam volume was corrected as *V*_foam,corrected_ = *V*_total_ – *V*_liq,corrected_, where *V*_total_ is the total volume measured by DFA. (see Supporting Information to see more detailed information about the correction and validation by an experiment.)

### 2.4 Definition of Foam Attributes

From the foam measurement, six foam attributes were extracted for subsequent modeling: maximum foam volume (*V*_foam max_), initial bubble count per unit area (BC_initial_), final bubble count per unit area (BC_final_), initial mean bubble radius (*R*_avg initial_), final mean bubble radius (*R*_avg final_), and bubble count half-life (*t*_BC 1/2_). From the corrected foam volume curve, *V*_foam max_ is defined as the foam volume at 50 s, i.e., 20 s after completion of foam generation. The 20 s delay is to remove the contribution of transient turbulence, which appears as a spike in the raw foam volume profile (Figure 2). Foam structure parameters were obtained by analyzing the bubble images collected during the rest period (Figure 3). BC_initial_, BC_final_, *R*_avg initial_, and *R*_avg final_ correspond to the bubble counts per unit area and mean bubble radius at 50 and 330 s, respectively. As an indicator of foam stability, *t*_BC 1/2_ is the time at which bubble count becomes half of BC_initial_ (Figure 4). Some body wash samples exhibit very high foam stability, so that *t*_BC 1/2_ is out of the measurement window. For those samples, *t*_BC 1/2_ is set to 400 s to represent high stability while maintaining numerical continuity for the regression models.

**Figure 3:**
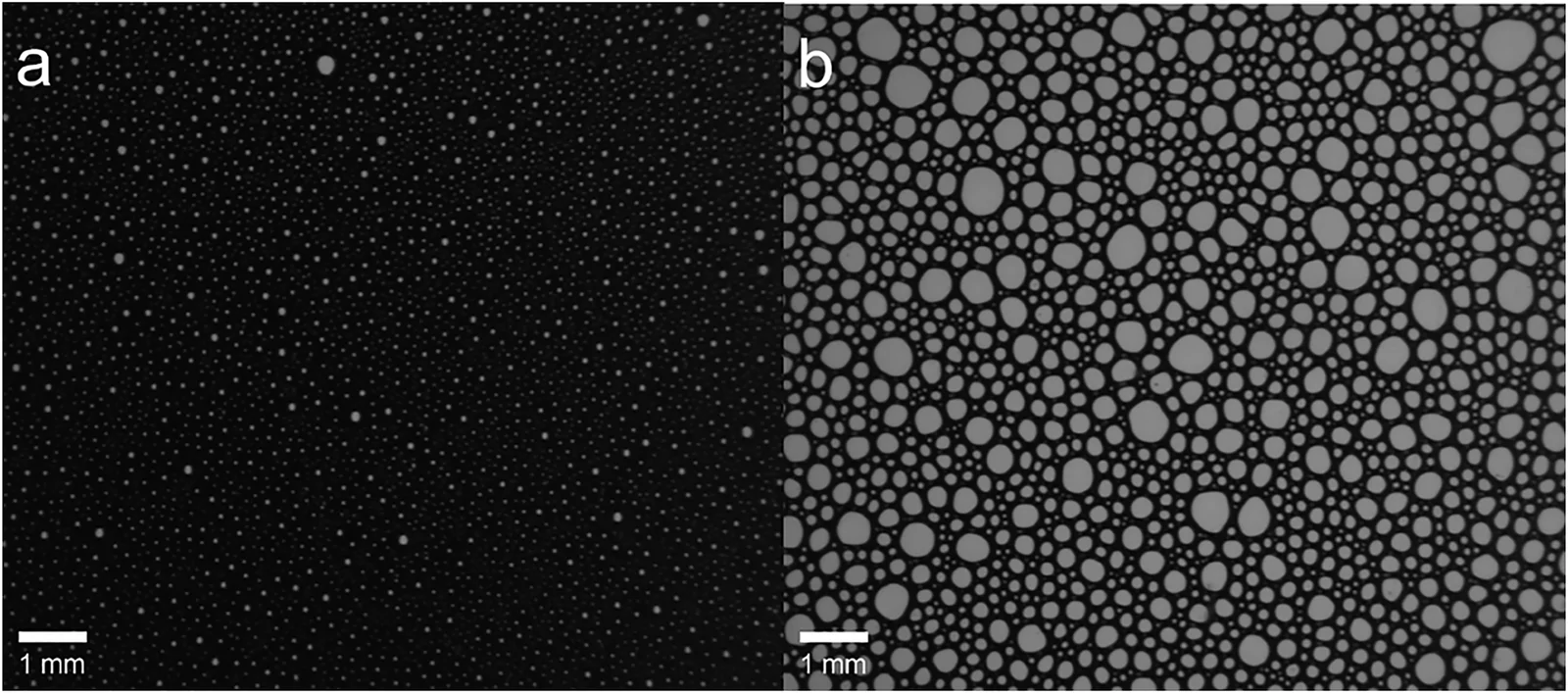
Foam images at (a) 50 s and (b) 330 s.

**Figure 4:**
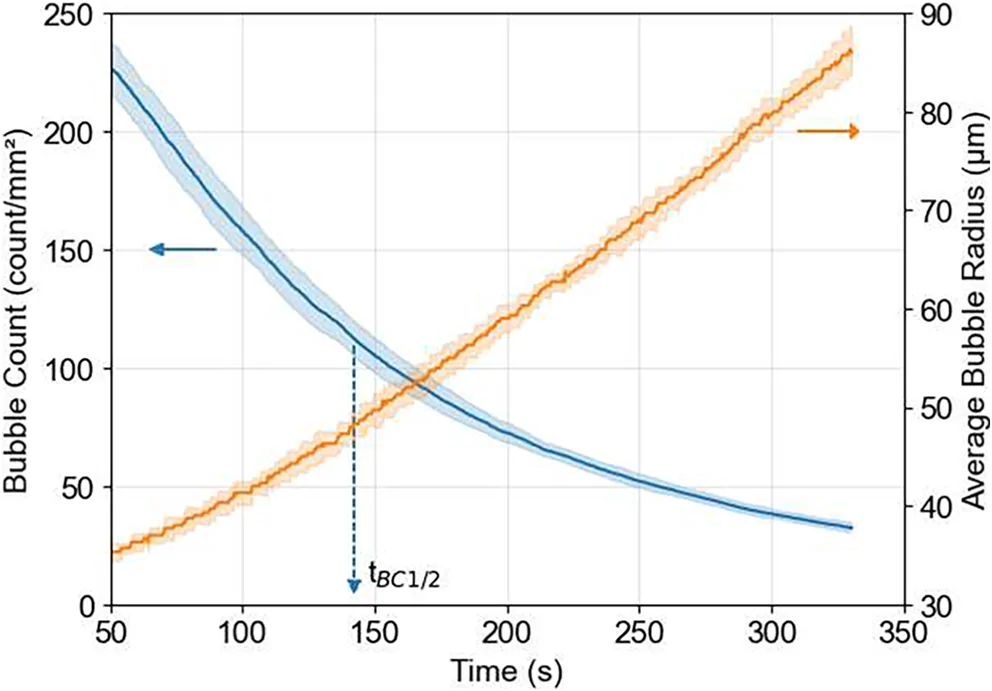
Evolution of bubble structure. The shaded area represents standard deviation.

### 2.5 Machine Learning Architectures

From the formulation matrix (Section 2.1), the net composition of a distinct ingredient by weight% was used as input features. The six foam attributes (Section 2.4) were used as target responses. Using Python’s scikit-learn library, five machine learning models were evaluated: K-Nearest Neighbors (KNN), Decision Tree (DT), Extra Trees (ET), Random Forest (RF), and Gradient Boosting Machines (GBM). The five algorithms deliberately span model families. KNN and DT are included as baseline approaches to demonstrate the performance necessity of ensemble models of RF, ET, and GBM.

KNN is an instance-based method that predicts the target values for a query point as the average of the k training samples closest to it in the feature space. Its performance degrades in high-dimensional settings because distances become less meaningful as dimensionality increases.

A DT recursively partitions the feature space by selecting, at each node, the feature and threshold that best separate the target values according to an error criterion. The resulting hierarchical structure is interpretable but prone to high variance: small changes in training data can produce substantially different trees, and deep trees tend to memorize training examples rather than learn generalizable patterns.^20^

On the contrary, ensemble tree models are known to be robust for handling high-dimensional, nonlinear data with complex ingredient interactions without requiring strict linearity assumptions.^21,22^ Compared to DT, they address the high variance of individual DTs by aggregating predictions from multiple trees. RF constructs an ensemble of trees each trained on a bootstrap sample of the data. RF decorrelates the trees further by considering only a random subset of features at each split. It uses the average prediction across all trees as the final prediction.^20,23^ ET follows a similar approach but introduces additional randomization by selecting split thresholds at random rather than optimizing them. GBM builds trees sequentially, with each new tree fitted to the residual errors of the current ensemble. This iterative refinement can reduce bias and variance, often yielding strong predictive performance.

The data set was randomly split into a training set (80%) and an independent test set (20%), which is a well-established baseline partition strategy in literature to ensure a sufficiently large validation set. The test set was strictly held out as an unseen validation vault, utilized solely for the final evaluation of model accuracy to completely eliminate the risk of data leakage or overoptimization bias. Using the GridSearchCV tool of scikit-learn library, hyperparameter optimization via grid search was conducted entirely within the 80% training partition. The grid search used 5-fold cross-validation within this partition, and the explored ranges are listed in Table S3 with the selected values in Tables S4–S6.

Predictive performance was then assessed by stratified 5-fold cross-validation on the full set of 545 formulations. For each attribute, the samples were divided into five quantile bins, and folds were assigned so that every fold of 109 formulations preserves the attribute’s distribution, including the bimodal bubble-structure attributes and the censored values at *t*_BC 1/2_ = 400 s. The procedure was repeated with five random shuffling seeds, yielding 25 validation folds per attribute, and identical folds were used for all five algorithms. Performance is reported as the mean and standard deviation over the 25 validation folds of the mean absolute error (MAE), root mean squared error (RMSE), and coefficient of determination (*R* ^2^), defined in (eqs 2–4).

2 $$\mathrm{MAE}=\frac{1}{N}\Sigma_{i}\left|x_{i}-{\mathrm{x̂}}_{i}\right|$$

3 $$\mathrm{RMSE}=\sqrt{\frac{1}{N}\Sigma_{i}{\left(x_{i}-{\mathrm{x̂}}_{i}\right)}^{2}}$$

4 $$R^{2}=1-\Sigma_{i}{\left(x_{i}-{\mathrm{x̂}}_{i}\right)}^{2}/\Sigma_{i}{\left(x_{i}-\mathrm{x̅}\right)}^{2}$$

where *N* is sample size, *x*_*i*_ is measured value for observation *i*, *x̂*_*i*_ is predicted value for observation *i*, and *x̅* is the mean of measured values. Using Python’s SHAP library, SHAP (SHapely Additive exPlanations) analysis was performed on the trained ET, GBM, and RF models.

## 3 Results and Discussions

### 3.1 Sample Statistics

To understand the overall foam characteristics of the studied body washes, the distribution of the six foam attributes across all 545 formulations is shown (Figure 5). *V*_foam max_ is approximately normally distributed. In contrast, both bubble size and bubble count exhibit bimodal distributions. Pairwise Pearson correlation coefficients between foam attributes are shown in the correlation matrix (Figure 6). *V*_foam max_ is largely uncorrelated with the other parameters, as indicated by low absolute correlation coefficients. In contrast, BC_initial_ and *R*_avg initial_ are strongly anticorrelated (*R* = −0.95), reflecting the intuitive expectation that, within a fixed field of view, smaller bubbles correspond to a larger count (Figure 3). BC_final_ and *R*_avg final_ are strongly correlated with *t*_BC1/2_, indicating that late-stage foam structure is closely linked to foam stability. Interestingly, the initial foam characteristics are only weakly correlated with foam stability as indicated by low correlation of *R*_avg initial_ with t_BC 1/2_ and of BC_initial_ with *t*_BC 1/2_. Given these cross-correlations, the following discussion will focus primarily on *V*_foam max_, BC_initial_, and t_BC1/2_ as representative foam attributes. Particularly, foam volume (*V*_foam max_) and foam density (BC_initial_) are primary interest of body wash design for consumer perception.

**Figure 5:**
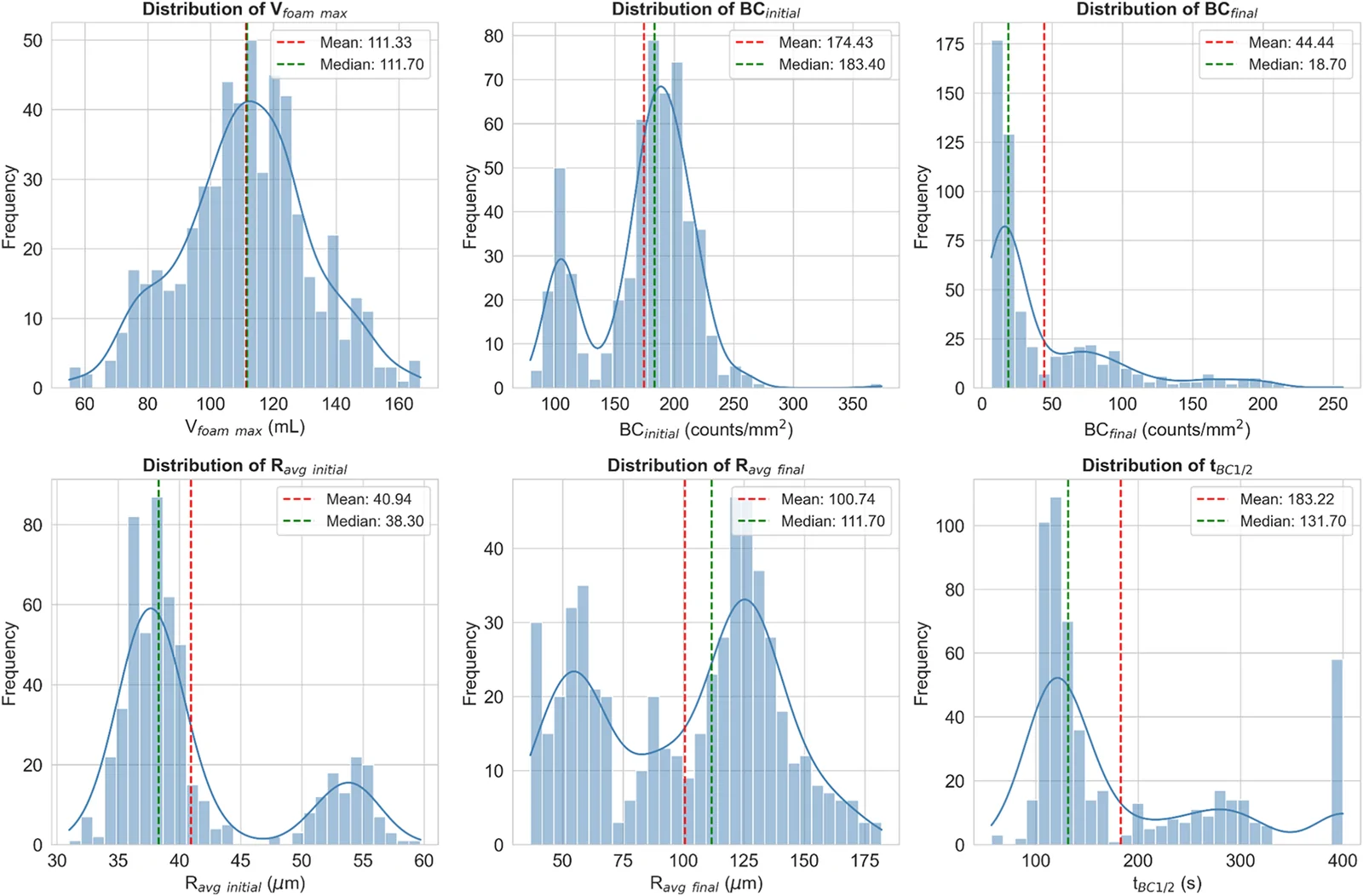
Histograms of the six foam attributes.

**Figure 6:**
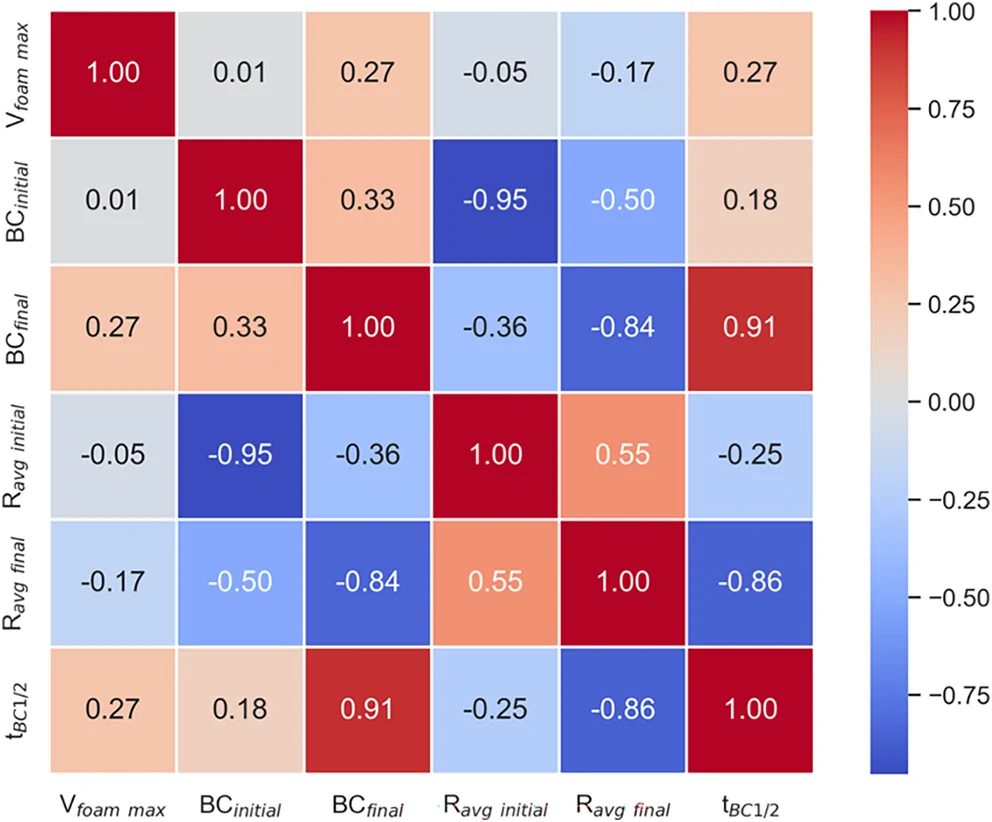
Correlation matrix of the six foam attributes. The numbers are Pearson correlation coefficients.

### 3.2 Validation of Machine Learning Models

The performance of the five ML algorithms was evaluated across all six foam attributes by repeated stratified 5-fold cross-validation (Table 1 and Figure 7 for *R* ^2^, Table 2 for MAE, and Table 3 for RMSE). Ensemble tree-based models (ET, RF, and GBM) outperformed the nonparametric KNN model and the single DT on every attribute, as evidenced by lower MAE and RMSE and higher *R* ^2^. As discussed in Section 2.5, KNN is not suitable for high-dimensional features. While KNN approaches the ensemble models on some attributes (e.g., cross-validated *R* ^2^ = 0.56 versus 0.61 to 0.64 for the ensembles on *V*_foam max_), its performance remains below the ensemble methods throughout, and the single DT trails further (*R* ^2^ = 0.27 for *V*_foam max_). Ensemble tree models outperform DT by reducing bias and variance. The mean *R* ^2^ per repeat varied by at most 0.05 across the five shuffling seeds for every model and attribute, indicating that the reported estimates are stable. Figure 8 plots out-of-fold predictions by the RF model against the actual measured values for all 545 formulations, where the dashed red line represents perfect prediction. Residual analysis of the out-of-fold predictions shows that the RF prediction is not biased across the bulk of the data (Figures S6 and S7). Residuals are centered at zero, with larger scatter and attenuation toward the mean only in the sparsely populated extremes of each attribute.

**Figure 7:**
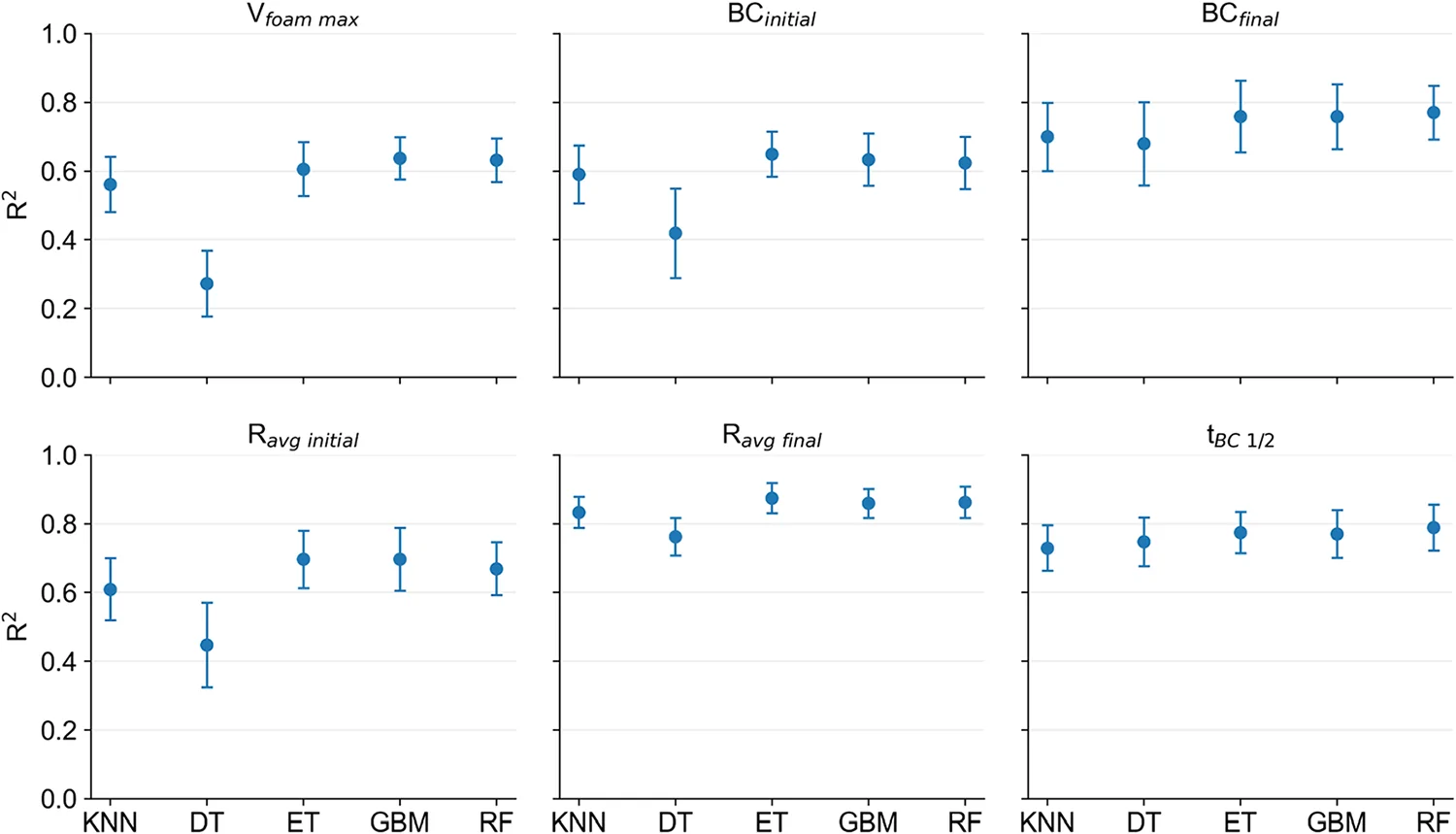
Cross-validated *R* ^2^ of the five ML models for each foam attribute. Markers are means and error bars are standard deviations over the 25 validations folds (stratified 5-fold cross-validation repeated with five shuffling seeds).

**Figure 8:**
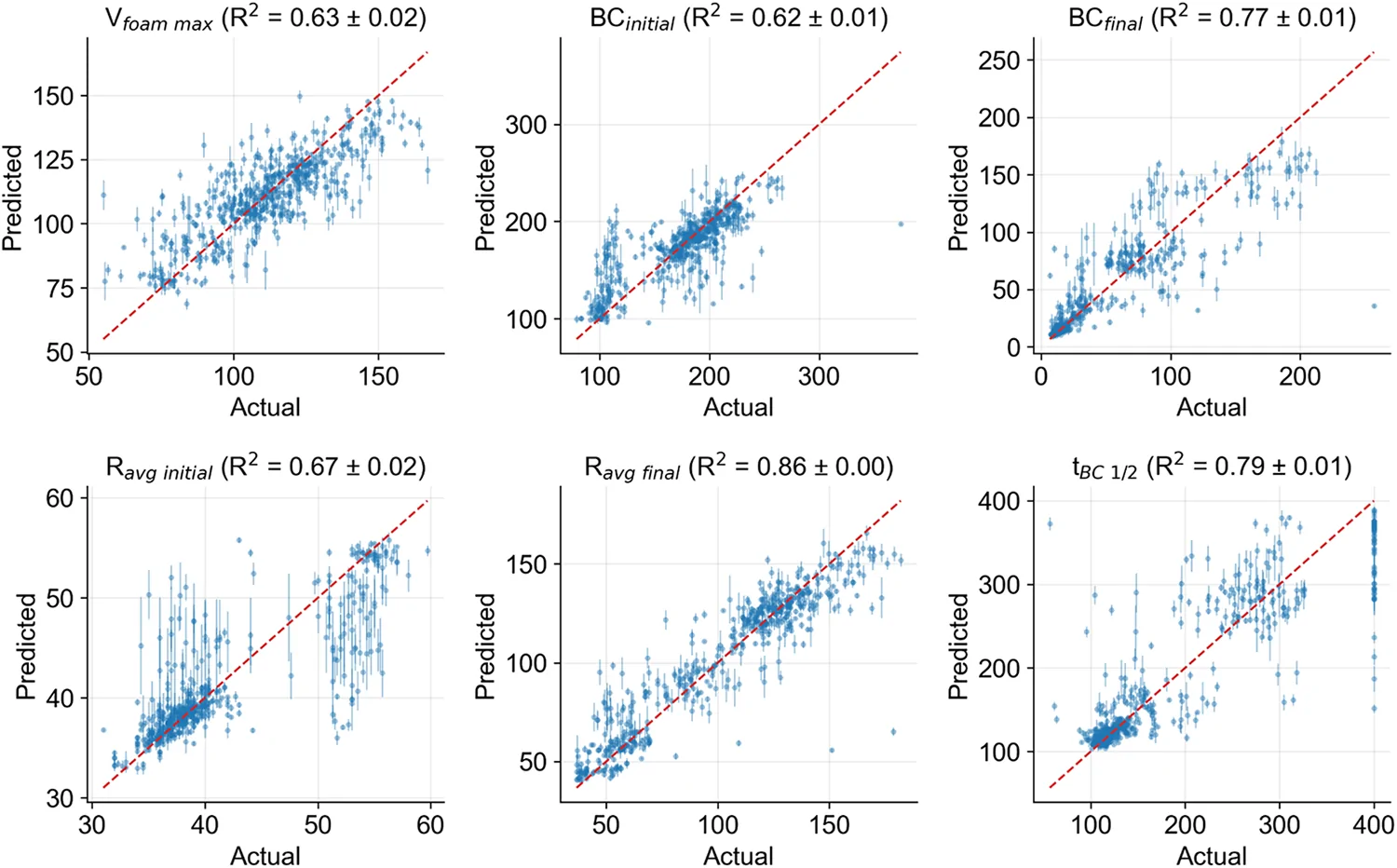
Out-of-fold predictions by the RF model versus actual values for all 545 formulations. In each repeat of the 5-fold cross-validation, every formulation is predicted once by the model that did not see it during training. Each point is the mean of the five out-of-fold predictions across the repeats, and the vertical error bar is the standard deviation across repeats. Panel titles report *R* ^2^ as mean ± SD over the five repeats. The red dashed line is the perfect prediction line.

**Table 1:** Cross-Validated *R* ^2^ for All Five Models (Mean ± SD over 25 Validation Folds from Stratified Five-Fold Cross-Validation Repeated with Five Shuffling Seeds)

	KNN	DT	ET	GBM	RF
*V*_foam max_	0.561 ± 0.080	0.273 ± 0.096	0.605 ± 0.078	0.637 ± 0.061	0.631 ± 0.063
BC_initial_	0.590 ± 0.084	0.419 ± 0.130	0.649 ± 0.065	0.633 ± 0.076	0.623 ± 0.076
BC_final_	0.699 ± 0.100	0.679 ± 0.121	0.758 ± 0.104	0.758 ± 0.094	0.770 ± 0.078
*R*_avg initial_	0.609 ± 0.090	0.447 ± 0.123	0.696 ± 0.083	0.696 ± 0.091	0.669 ± 0.077
*R*_avg final_	0.833 ± 0.045	0.762 ± 0.055	0.874 ± 0.044	0.859 ± 0.042	0.862 ± 0.045
*t*_BC1/2_	0.729 ± 0.066	0.747 ± 0.071	0.774 ± 0.060	0.770 ± 0.070	0.789 ± 0.067

**Table 2:** Cross-Validated MAE for All Five Models (Mean ± SD over 25 Validation Folds)^a^

	KNN	DT	ET	GBM	RF
*V*_foam max_	9.89 ± 1.02	13.51 ± 1.16	9.42 ± 0.86	9.23 ± 0.76	9.21 ± 0.77
BC_initial_	17.47 ± 1.72	21.84 ± 2.75	16.06 ± 1.57	16.72 ± 1.85	16.74 ± 1.65
BC_final_	13.23 ± 2.08	14.10 ± 1.98	11.47 ± 1.94	11.72 ± 1.58	11.67 ± 1.12
*R*_avg initial_	2.49 ± 0.26	3.25 ± 0.34	2.21 ± 0.23	2.22 ± 0.23	2.21 ± 0.21
*R*_avg final_	9.95 ± 0.97	12.61 ± 0.90	8.67 ± 0.85	9.43 ± 0.82	9.32 ± 0.80
*t*_BC1/2_	29.40 ± 2.65	28.85 ± 2.49	26.19 ± 2.27	26.91 ± 2.85	25.57 ± 2.57

a MAE carries the units of each attribute (mL for *V*_foam max_, counts/mm^2^ for the BC_initial_ and BC_final_, μm for the *R*_avg initial_ and *R*_avg final_, and s for *t*_BC 1/2_).

**Table 3:** Cross-Validated RMSE for All Five Models (Mean ± SD over 25 Validation Folds)^a^

	KNN	DT	ET	GBM	RF
V_foam max_	13.53 ± 1.38	17.45 ± 1.32	12.81 ± 1.24	12.32 ± 1.14	12.40 ± 1.12
BC_initial_	26.41 ± 3.51	31.37 ± 4.11	24.46 ± 2.97	24.98 ± 3.42	25.30 ± 3.20
BC_final_	25.53 ± 4.91	26.26 ± 5.70	22.78 ± 5.63	22.74 ± 4.54	22.21 ± 3.47
*R*_avg initial_	4.17 ± 0.44	4.97 ± 0.55	3.67 ± 0.45	3.67 ± 0.50	3.84 ± 0.42
*R*_avg final_	15.20 ± 2.25	18.16 ± 2.02	13.10 ± 2.41	13.93 ± 2.16	13.77 ± 2.29
*t*_BC1/2_	50.03 ± 6.25	48.16 ± 6.53	45.59 ± 5.99	45.85 ± 6.98	43.88 ± 6.94

a RMSE carries the units of each attribute (mL for *V*_foam max_, counts/mm^2^ for the BC_initial_ and BC_final_, μm for the *R*_avg initial_ and *R*_avg final_, and s for *t*_BC 1/2_).

### 3.3 Feature Importance Analysis

To interpret the trained models and identify the most influential formulation variables, we computed SHAP analysis on the trained models by ET, GBM, and RF. SHAP values of the features (ingredients in this study) represent the importance of them in the trained models.^24^ Although SHAP analysis provides the interpretability of the trained models, as ML is not based on the first principle of chemistry, we found that it is challenging to interpret some of the selected influential ingredients. Figure 9 shows the five most important ingredients for three foam attributes from the ML models, ranked by absolute mean SHAP value. Each data point represents each test sample, and the color represents the value of the feature: red is high and blue is low. If red data points are on positive SHAP values, it means that a higher level of the feature increases the prediction value. If blue data points are on negative SHAP values, it means that a lower level of the feature decreases the prediction value. If the data points are near zero values, it means that the feature has little influence on the attribute. Each model assigns different relative weights to individual ingredients, reflecting model-specific interactions and nonlinearities. To build a solid understanding, we will focus on ingredients that appear among the top contributors across multiple models. DT and KNN models were excluded from the consensus analysis because of their relatively low *R* ^2^ values across the target responses.

**Figure 9:**
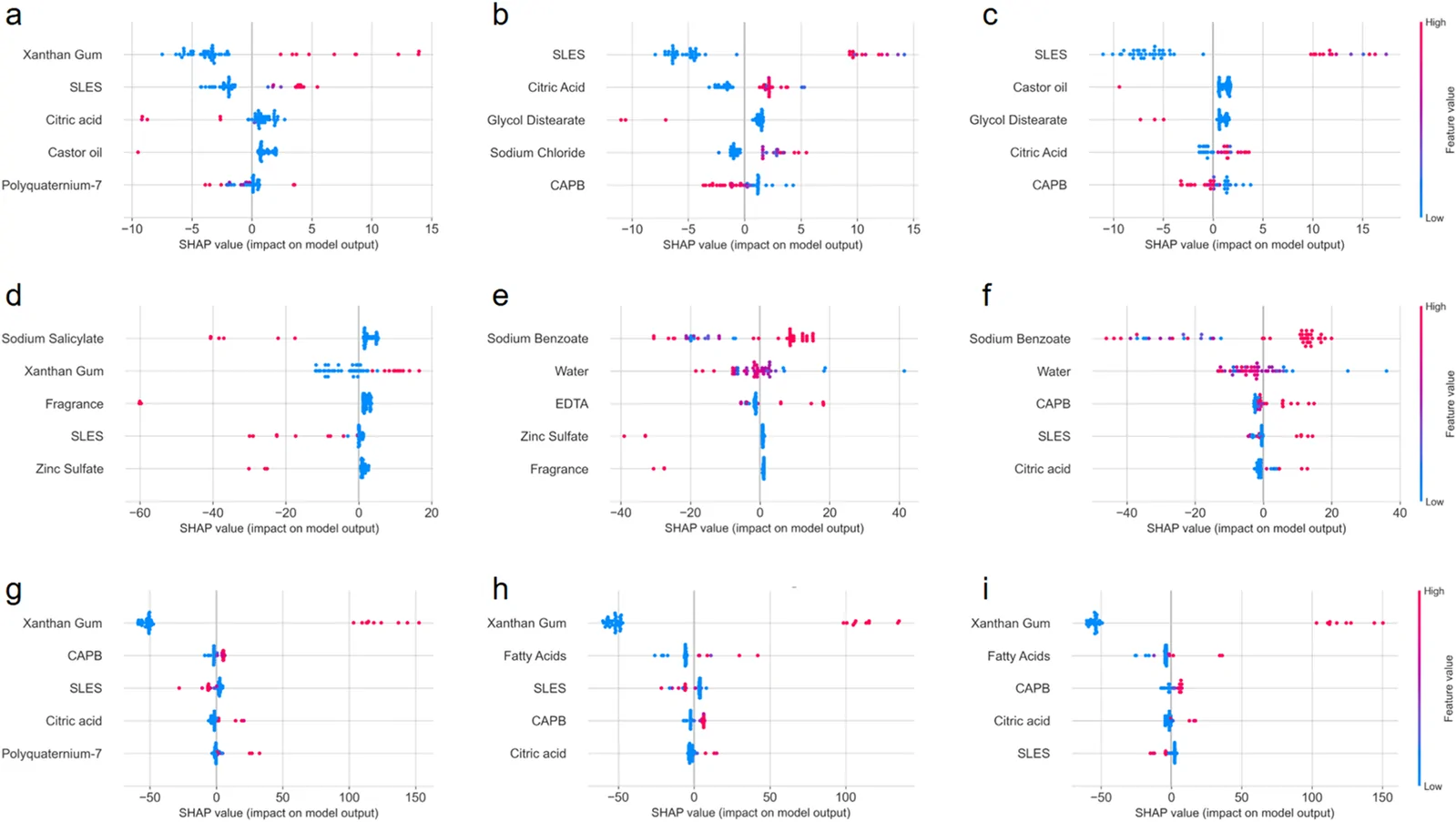
Feature importance comparison for (a–c) *V*_foam max_, (d–f) BC_initial_ and (g–i) *t*_BC 1/2_ between different ML models of (a, d, g) Extra Trees, (b, e, h) Gradient Boosting Machines and (c, f, i) Random Forest.

For *V*_foam max_, SLES (sodium laureth sulfate), CAPB (cocamidopropyl betaine), citric acid, castor oil, and glycol distearate emerge as key ingredients. The combination of SLES and CAPB is the most used surfactant system of the studied samples, and it is also widely used in personal care products. When amphoteric CAPB is introduced to anionic SLES, it reduces charge repulsion between SLES molecules, which leads to increased product viscosity as well as increased viscoelastic modulus of liquid–air interface. Because of these factors, SLES and CAPB are well-known for their foam boosting effect when they are used together.^4,25^ However, the SHAP analysis shows a negative effect of CAPB on foam volume. For effective foam generation, the balance between facile air entrapment and high stability of the entrapped air is needed. If surface viscoelastic modulus or solution viscosity is too small, air is easily introduced to the system, but bubbles easily collapse. On the contrary, if those parameters are too high, bubbles are hard to generate.^2,26^ It could be that within the given data, CAPB decreases foam volume at the cost of foam stability. Citric acid is used in body wash to adjust pH, and it is unclear why it appears influencing foam volume. In the tested samples, most body washes are acidic, while soap-based body washes are alkaline to neutralize fatty acids. It may indicate the former foams more than the latter. Castor oil and glycol distearate serve as opacifiers in body wash, and they are observed to decrease foam volume, likely because the solid particles often reduce foamability by increasing viscosity and surface viscoelasticity.^6^

It is not straightforward to interpret SHAP analysis for BC_initial_. Sodium benzoate and sodium salicylate are preservatives for the body washes. It likely indicates that certain brands with those preservatives have different foam density from the others.

For foam stability (*t*_BC1/2_), xanthan gum is selected as the most influential ingredient, followed by fatty acids, SLES, and CAPB. It has been reported that polysaccharide such as xanthan gum enhances foam stability by increasing solution viscosity.^25,27^ Fatty acids are also well-known as foam stabilizers in the system of SLES + CAPB by increasing viscoelasticity of the air–liquid interface, which slows down water drainage.^2,28,29^ CAPB would similarly enhance foam stability because with SLES it increases liquid viscosity and viscoelasticity of the interface.^2,30^

### 3.4 Prediction of New Formula

To demonstrate the practical application of the ML model, we constructed a formulation design space comprising all ingredients that appear in Figure 9 except fragrance, which are top influential ingredients selected by ET, GBM, and RF. The concentration ranges of each ingredient were defined from its fifth to 95th percentile levels observed across the full data set. Within this constrained design space, 100 candidate formulations were generated using Latin Hypercube Sampling, which selects near-random samples in the 13-dimensional design space while maintaining good coverage of the input range. The remaining fraction of each formulation was balanced with water. The trained RF model was then used to predict the foam attributes of each formulation.

Among the 100 formulations, Figure 10 highlights three formulations: one with the highest *V*_foam max_, one with the highest BC_initial_, one with the highest *t*_BC 1/2_. The radar plot is normalized to the maximum value of each attribute across the candidate data set. Formulation 2 differs markedly from Formulation 1 and 3 in foam density. Relative to Formulation 1, Formulation 2 exhibits ∼10% lower *V*_foam max_, but substantially higher foam density (BC_initial_). In foam stability (*t*_BC 1/2_), all formulations are similar. These results suggest that Formulation 2 would be a suitable choice when high foam density is prioritized over foam volume.

**Figure 10:**
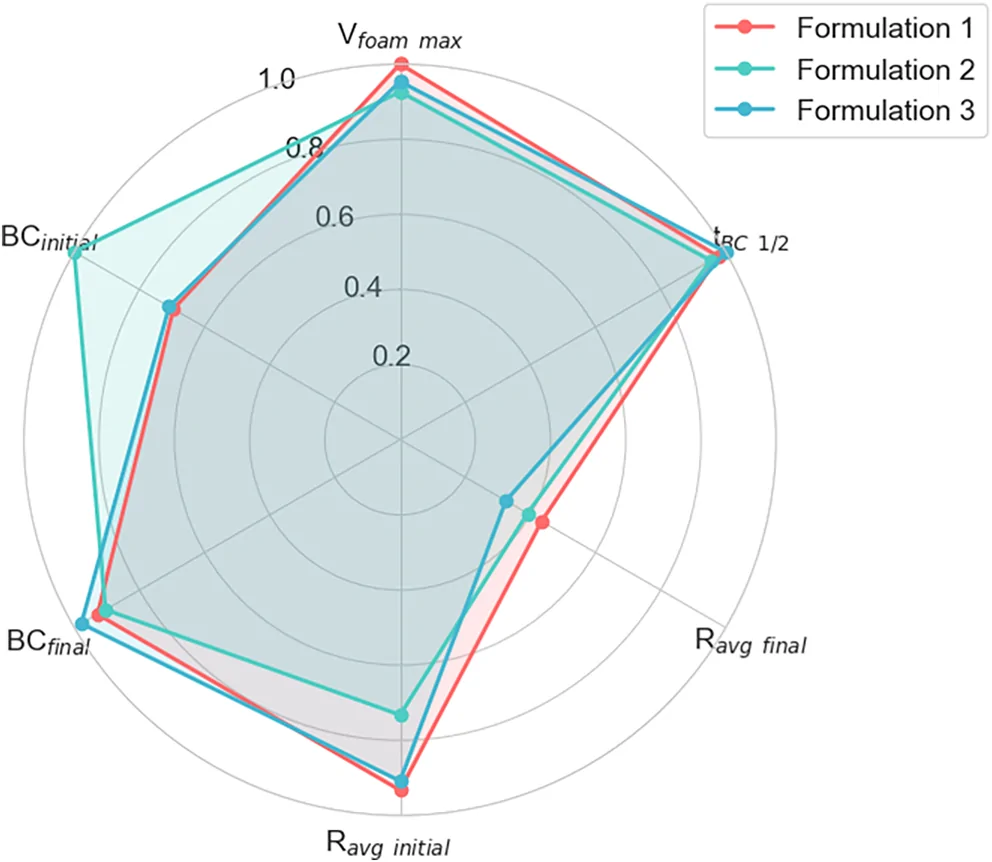
Predicted foam attributes of new formulation.

## 4 Conclusion

This study demonstrates the potential of machine learning for accelerated formulation development by predicting the foaming properties of body wash products. The formulation principles and modeling framework, which focus on common surfactants and polymers such as SLES, CAPB, and xanthan gum, are broadly applicable to other liquid personal care technologies like hand and face wash. A key prerequisite for this work was the construction of a high-quality data set linking detailed formulation composition to quantitatively measured foam attributes. By systematically compiling formula information and DFA-derived foam parameters into an ingredient–property matrix, we enabled the training and evaluation of multiple ML models. Five algorithms—DT, ET, RF, GBM, and KNN—were benchmarked. Ensemble methods consistently outperformed the single DT and KNN. Analysis of the foam attributes revealed that *V*_foam max_ is largely uncorrelated with the other foam parameters such as foam density, bubble size and foam stability, as indicated by low correlation coefficients. SHAP-based consensus feature analysis revealed that SLES, CAPB, opacifiers, fatty acids and xanthan gum are the dominant ingredients governing foam volume, initial bubble structure, and foam stability. Although SHAP analysis improves the interpretability of the tree-based models, some of the identified relationships remain challenging to rationalize from a purely mechanistic standpoint—for example, the negative effect of CAPB on foam volume and the apparent influence of preservatives on bubble density. These findings highlight both the value and the limitations of data-driven models for uncovering structure–property relationships in complex formulations. Overall, the trained ML models are capable of predicting the foamability of full, multicomponent commercial formulations.

### 4.1 Limitations

The applicability of the trained models is bounded by the training data. Predictions are reliable within the composition space spanned by the 545 formulations and the 362-ingredient library; ingredients outside this library cannot be represented, and extrapolation beyond observed concentration ranges is not supported. Initial-stage attributes (BC_initial_ and *R*_avg initial_) carry wider prediction uncertainty (cross-validated *R* ^2^ near 0.6) than late-stage attributes. Foam stability values above the 400 s measurement window are censored, so distinctions among very stable foams are not resolved. All targets reflect one measurement protocol (DFA at 5 wt.% dilution with a fixed agitation sequence), and transfer to other test conditions has not been evaluated.

## Supplementary Material



## Notes

Additional figures, tables, and notes; reasons for and the method of DFA foam volume correction; scheme of foam height detection by DFA; foam volume profiles and the corrected profiles; validation of the correction method; hyper parameter optimization grid search for each ML model; selected hyperparameters for tree-based models; residual analysis for RF prediction models (PDF)

## §

Amazon Web Services, 410 Terry Avenue North, Seattle, Washington 98109, United States

## ∥

NVIDIA Corporation, 2788 San Tomas Expressway, Santa Clara, California 95051, United States

## ⊥

Kenvue Inc., 1 Kenvue Way, Summit, New Jersey 07901, United States

## #

Church and Dwight Co. Inc., 469 North Harrison St., Princeton, New Jersey 08540, United States
