# Supplementary material for: Predicting Foam Characteristics of Body Wash Products Using Machine Learning
Source: ACS Omega. 2026 Oct 24;11(43):66139–48. doi: 10.1021/acsomega.6c03163 (PMC13737078; doi:10.1021/acsomega.6c03163)
Supplement: Supplementary file 1 [file ao6c03163_si_001.pdf]

Supporting Information

Predicting Foam Characteristics of Body Wash Products using Machine Learning

Jonghun Lee[1*], Sivaramakrishnan Swamyna[2], Akshay Adithya[2], Viktor Dubovoy[1], Paul Thomson[1], Detravious J. Brinkley[2], Chibuzor J. Okocha[2], Christan Grant[2]

[1] Colgate-Palmolive Co., 909 River Rd., Piscataway, New Jersey 08854, U.S.A.
[2] Department of Computer & Information Science & Engineering, University of Florida, 1889 Museum Road, Gainesville, FL 32611, U.S.A.

*Corresponding author: jonghun_lee@colpal.com

1. Foam Volume Correction

When foam is generated, a liquid layer and a foam layer are formed. By difference in transmission of blue light through the layers, Dynamic Foam Analyzer (DFA) from Kruss automatically detects liquid height and foam height (Figure S1). For example, the air and liquid layers are transparent, while the foam layer in the middle is not. It detects the interfaces where the transmission behavior changes. By analyzing these height profiles, DFA draws foam characteristics such as $V_{foam\ max}$ (maximum foam volume), FVS (ratio of foam volume to the reference foam volume) and FLS (ratio of volume of liquid bound to foam to that of reference value). The accuracy of these values depend on the correct reading of liquid, foam, and total heights. However, blue light is blocked by the stirrer on the bottom. DFA fails to correctly measure the liquid height when the liquid height is below the height of the stirrer, which leads to wrong analysis on those characteristic values. This supporting information shows how to correct the liquid profile by fitting it with an exponential-plateau model to obtain the right foam profiles and the correct characterization, and it is validated by an experiment.

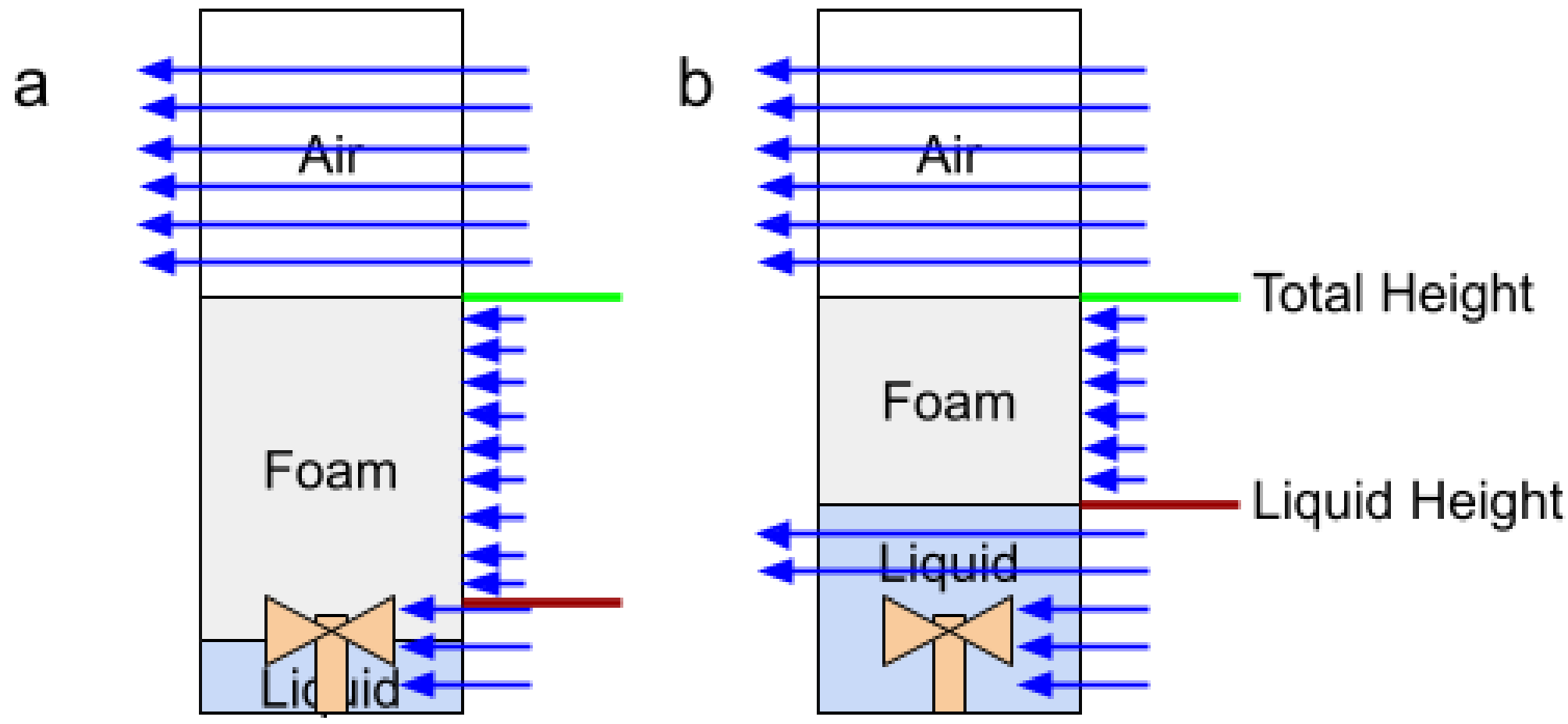

Figure S1. Schematic of foam height profile measurement by DFA that uses transmission of blue lights (a) when the liquid line is below the stirrer and (b) when the liquid line is above it. The blue rays indicate the light path way through the column. The green line indicates total height, and the red line indicates the liquid height measured by DFA.

DFA experiments generally consist of two parts of foam generation and foam decaying. During the foam generation step, the samples are stirred following a set protocol. During the foam decaying step, the generated foam is left unagitated for a period of time. ADVANCE, the DFA software, characterizes foam properties by using data from the foam decaying step. Key parameters are

$$V_{foam\ max} = V_{total\ max} - V_{liquid\ at\ foam\ max}$$

$$FVS = \frac{V_{foam,\ current\ time}}{V_{foam,\ reference\ time}}$$

$$FLS = \frac{V_{liquid\ bound\ to\ foam,\ current\ time}}{V_{liquid\ bound\ to\ foam,\ reference\ time}}$$

Experiments

A commercial body wash was diluted to 5% by weight in DI water. 50 mL of sample was used. It was stirred for 5 sec followed by a 3 sec break. This stir-stop repeated 6 times, followed by 300 sec rest.

For a validation experiment where manual reading of liquid height was performed, the tape measure was attached on one side of the column, and liquid height was measured when it reached every graduate.

Result

When stirring mode is used, as the stirrer blocks blue light, liquid lines below 27.0 mm cannot be measured (Figure S2). For the first 83 seconds after stirring stops, the liquid height curve is 27.0 mm, and the foam height is about 76 mm. However, by observation, when stirring stopped, initially foam filled the entire column, and the liquid line grew from 0 mm.

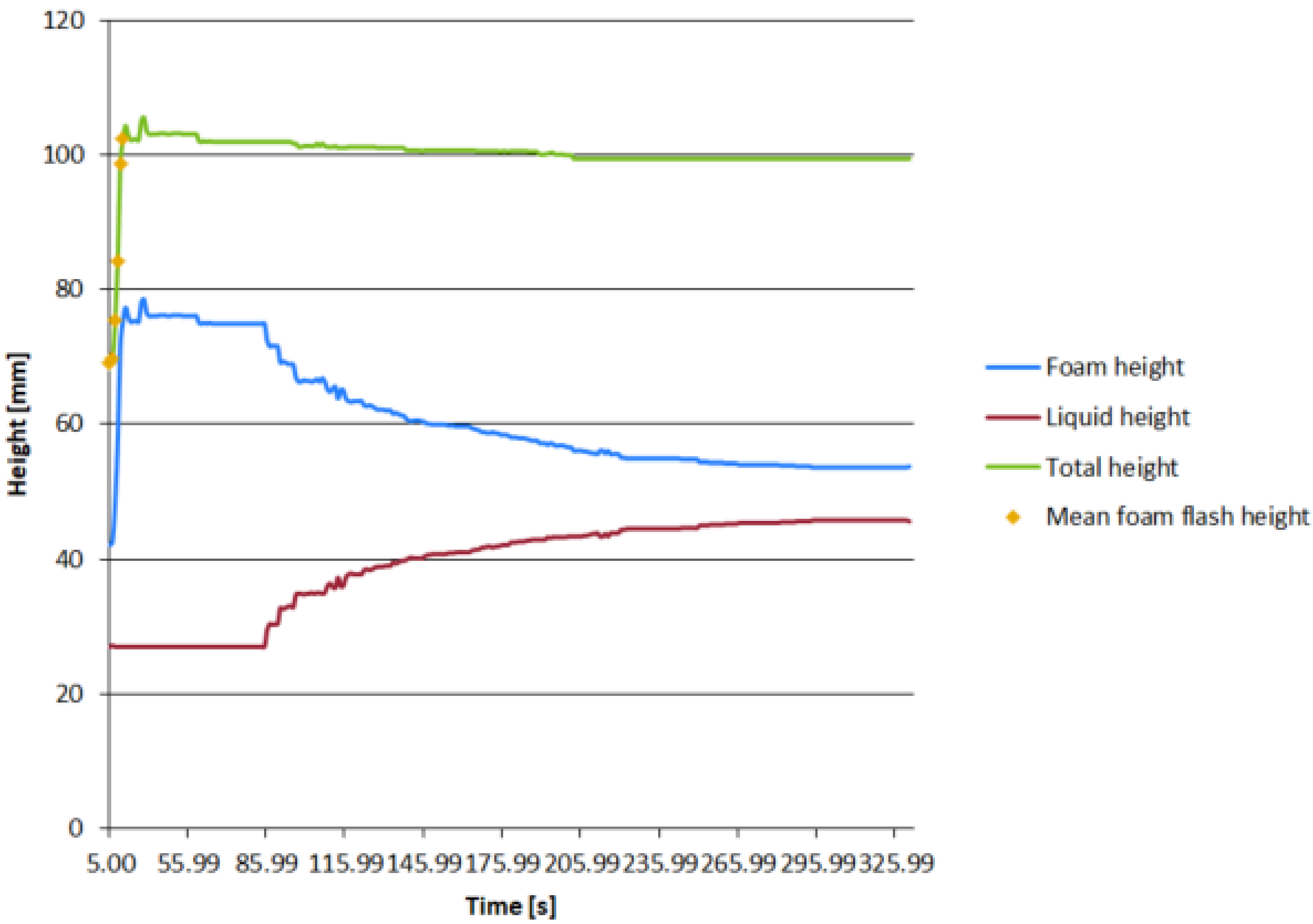

Figure S2. Height profile of stirring mode test provided by the DFA software (Advance)

To estimate the liquid volume profile in the early stage, the liquid volume curve is fitted with the exponential plateau model (eq. S1). , in the range after the liquid height begins to increase (t_{onset}). For example, in Figure 2S, t_{onset} = 83 s, and fitting was made where t > t_{onset}.

$$v = v_m - (v_m - v_0)e^{-k(t-t_0)} \qquad \text{(S1)}$$

where v_m is plateau volume, v_0 is initial volume, k is rate constant, t_0 is the true onset time point, where liquid grows from 0 mL. The fitting range is set as follows. First, the onset time (t_{onset}) was found where the liquid line grows from the measured graph. For example, the onset time (t_{onset}) is 83 s in Figure S2. The fitting range was set to t > t_{onset}. The new liquid volume is

$$v_{liquid} = 0, \qquad if\ t < t_0$$

$$v_{liquid} = fitted\ line, \qquad if\ t_0 < t < t_{onset}$$

$$v_{liquid} = measured\ v_{liquid}\ by\ DFA,\ if\ t > t_{onset}$$

With the new v_{liquid}, v_{foam} is simply obtained by $v_{foam} = v_{total} - v_{liquid}$. With the new profiles, foam characteristic parameters are re-calculated (Table S1)

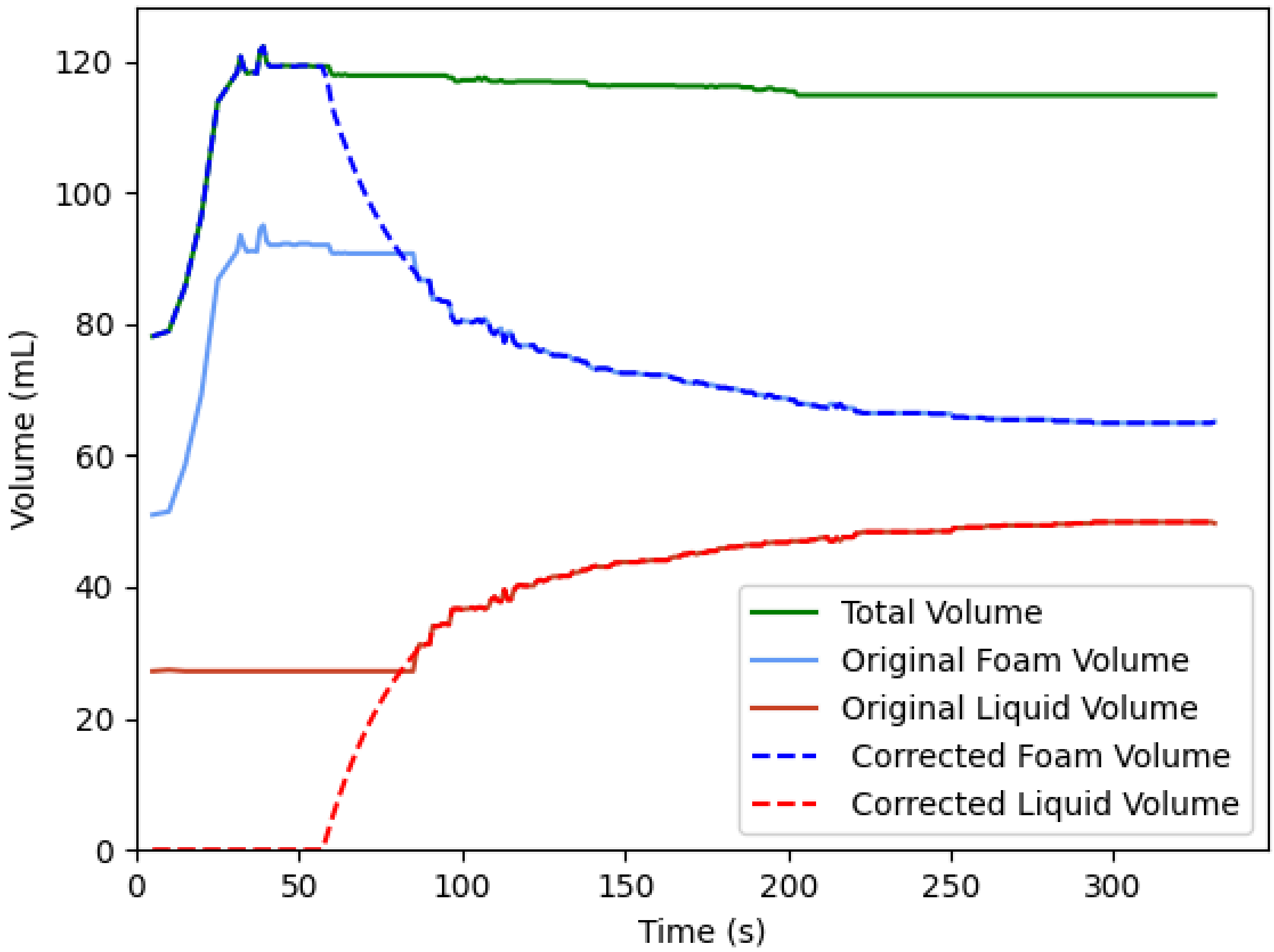

Figure S3. Corrected liquid and foam volume profiles plotted with original curves measured by DFA.

Table S1. Height - Results

Name	Value	Corrected Value	Unit
t_{ref}	50.00	50.00	s
$V_{foam\ max}$	92.2	119.3	mL
$V_{liquid\ foam\ max}$	22.8	50.0	mL
FC	--		
MD	0.2	0.4	
ER	4.0	2.4	
$t_{FVS\ 50\%}$	--		s
$t_{FLS\ 75\%}$	91.0	66.0	s
$t_{FLS\ 50\%}$	113.0	79.0	s
HRV	51.0	30.0	s
$\overline{T}$	--		°C

Validation of Exponential Fit

To validate the correction method, the height increase was manually measured (Figure S4). A tape measure was attached to the DFA column. When the liquid-foam border line reaches readable graduates, the remaining time was recorded, which is provided real-time by the DFA software. The remaining time was converted to actual experimental time by Experiment time = 300 s - Remaining time + 30 s (Table S2). The height is scaled to match the measured liquid volume and plotted in Figure S5. It confirms that the manual reading is well overlaid on the measured liquid line and fitted part.

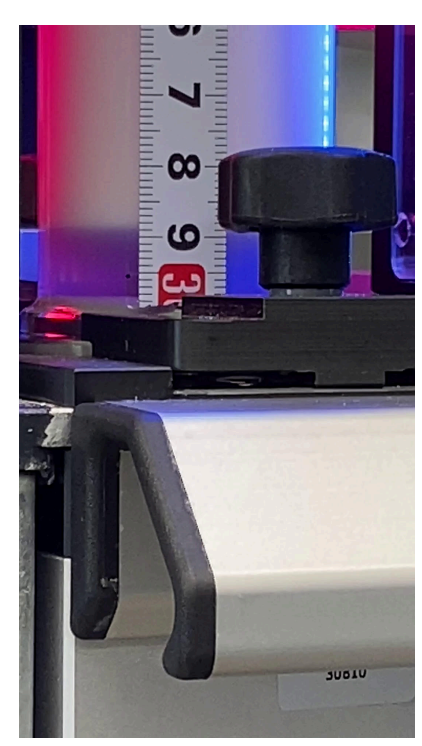

Figure S4. Manual measurement of liquid height.

Table S2. Manual Liquid-Foam Line Reading

Liquid Height (mm)	Remaining time (s)	Experiment time (s)
4	275	55
7	269	61
9	263	67
11	254	76
14	248	82
15	245	85
16	240	90
17	235	95
18	226	104
19	218	112
20	209	121
21	201	129
22	192	138
24	166	164
25	156	174
26	133	197
27	113	217
28	94	236
29	58	272
30	27	303

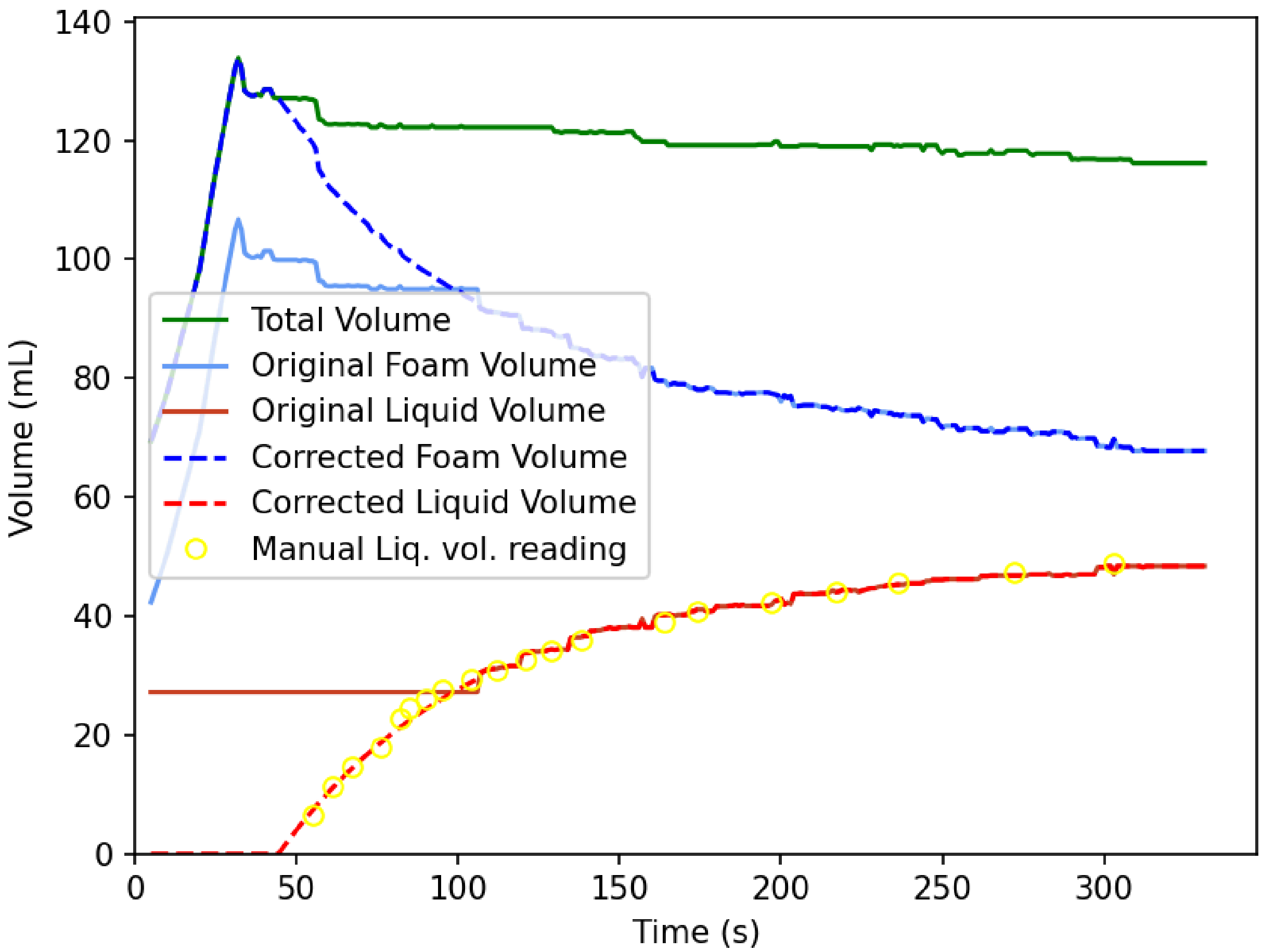

Figure S5. The validation of the correction method by overlaying with manual measurement.

2. Hyper Parameter Optimization

The grid search used GridSearchCV with five-fold cross-validation within the 80% training partition.

Table S3. Hyper Parameter Optimization Grid Search

Model	Hyperparameter	Description	Explored Grid Values
**K-Nearest Neighbors (KNN)**	N_neighbors	Number of nearby instances	[3, 5, 10, 20, 40]
	weights	Weight function used in prediction	['uniform', 'distance']
	metric	Distance metric	['euclidean', 'manhattan']
**Decision Tree (DT)**	max_depth	Maximum depth of the tree	[None, 10, 20, 30, 40, 50]
	min_samples_split	Minimum samples to split an internal node	[2, 5, 10, 20, 40, 80]
	min_samples_leaf	Minimum samples required at a leaf node	[1, 2, 4, 8, 16, 32]
**Extra Trees (ET)**	n_estimators	Number of trees in the forest	[50, 100, 200]
	max_depth	Maximum depth of the individual trees	[None, 10, 20]
	min_samples_split	Minimum samples to split an internal node	[2, 5, 10]

**Random Forest (RF)**	n_estimators	Number of trees in the forest	[50, 100, 200]
	max_depth	Maximum depth of the individual trees	[None, 10, 20]
	min_samples_split	Minimum samples to split an internal node	[2, 5, 10]
	min_samples_leaf	Minimum samples required at a leaf node	[1, 2, 4]
**Gradient Boosting (GBM)**	n_estimators	Number of boosting stages	[50, 100, 200]
	learning_rate	Step size shrinkage to prevent overfitting	[0.01, 0.1, 0.3]
	max_depth	Maximum depth of individual estimators	[3, 4, 5]
	min_samples_split	Minimum samples to split an internal node	[2, 5, 10]
	min_samples_leaf	Minimum samples required at a leaf node	[1, 2, 4]
	subsample	Fraction of samples for fitting individual trees	[0.8, 1.0]

Table S4. Selected hyperparameters for RF (tuned per attribute).

Attribute	n_estimators	max_depth	min_samples_split	min_samples_leaf
$V_{foam\ max}$	200	None	2	1

$BC_{initial}$	200	20	2	2
BC_{final}	50	None	2	2
$R_{avg\ initial}$	50	None	2	4
$R_{avg\ final}$	200	None	2	2
$t_{BC\ 1/2}$	200	20	10	2

Table S5. Selected hyperparameters for GBM (tuned per attribute).

Attribute	n_estimators	learning_eate	max_depth	min_samples_split	min_samples_leaf	subsample
$V_{foam\ max}$	100	0.1	3	5	2	0.8
$BC_{initial}$	50	0.3	3	2	4	0.8
BC_{final}	200	0.1	5	2	2	0.8
$R_{avg\ initial}$	200	0.1	3	2	4	1.0
$R_{avg\ final}$	100	0.3	3	10	4	1.0
$t_{BC\ 1/2}$	50	0.1	4	2	2	1.0

Table S6. Selected hyperparameters for the multi-output models (one configuration for all six attributes).

Model	n_estimators	max_depth	min_samples_split	min_samples_leaf	n_neighbors	weights	metric
ET	100	20	10	N/A	N/A	N/A	N/A
DT	N/A	None	80	1	N/A	N/A	N/A
KNN	N/A	N/A	N/A	N/A	5	distance	manhattan

3. Residual Analysis

Even spread of scatter points near zero residual line in the residual scatter plot indicates the no systematic bias in these RF prediction models (Figure S6). Similarly, the normal distribution of residuals near zero residual indicates the unbiased and efficient prediction (Figure S7).

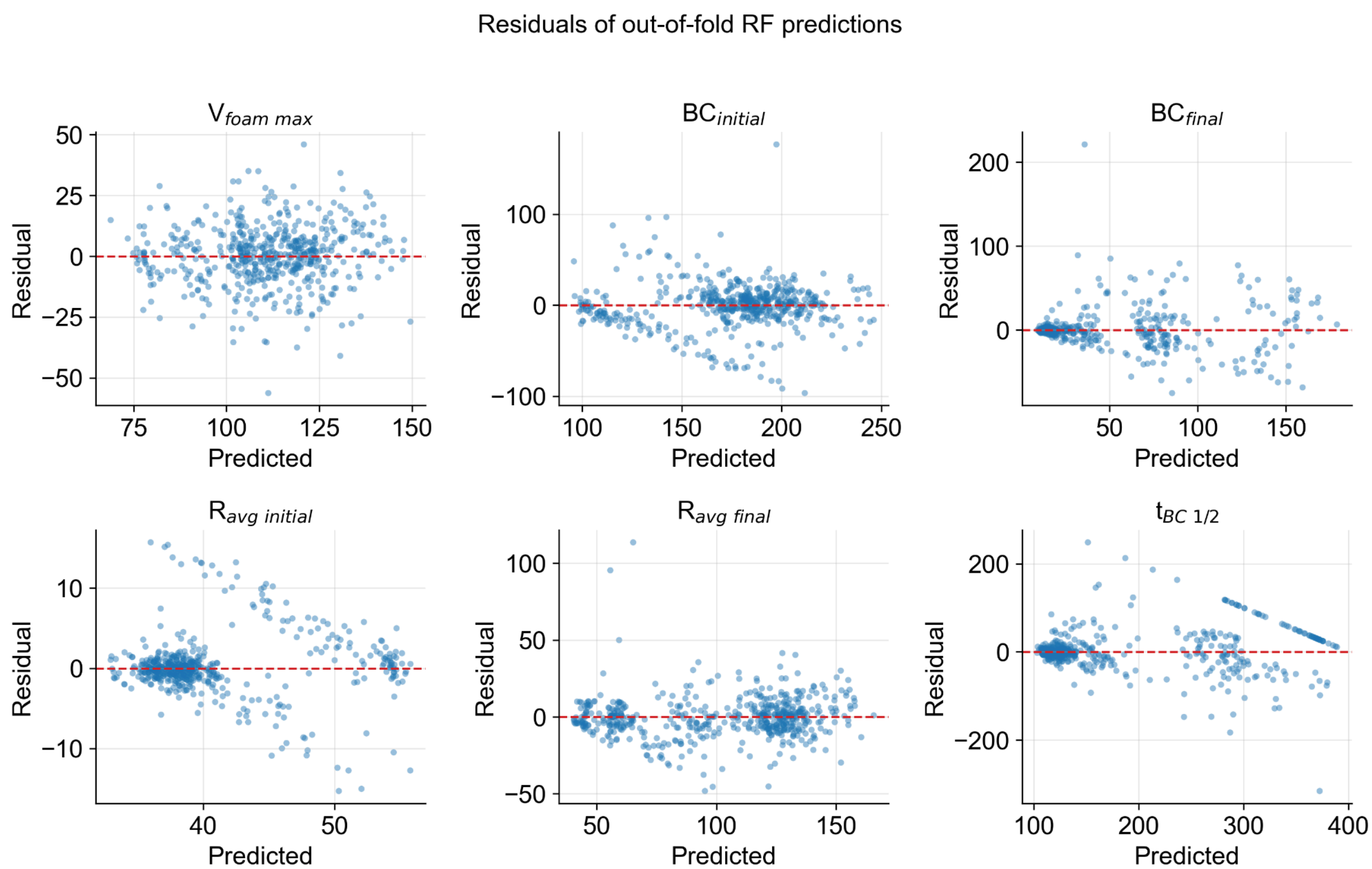

Figure S6. Residuals (actual − predicted) versus predicted values for the RF model, computed from the mean out-of-fold predictions of the repeated stratified five-fold cross-validation. The diagonal streak near 400 s in the $t_{BC\ 1/2}$ panel corresponds to samples censored at the 400 s measurement window.

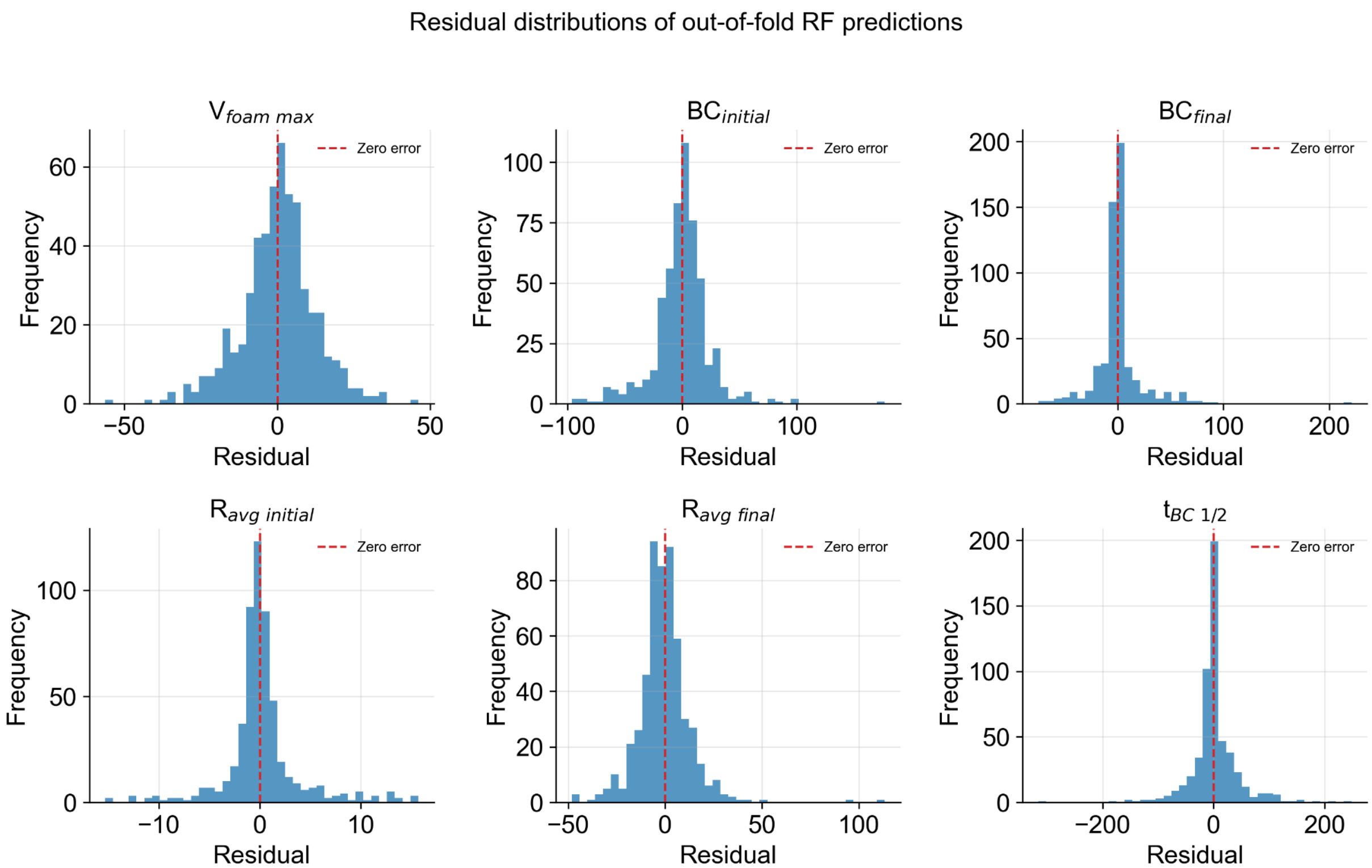

Figure S7. Residual distributions for the RF model from out-of-fold predictions. The dashed line marks zero error.
